# At the Forefront of the Mucosal Barrier: The Role of Macrophages in the Intestine

**DOI:** 10.3390/cells9102162

**Published:** 2020-09-24

**Authors:** Barbara Ruder, Christoph Becker

**Affiliations:** Department of Medicine 1, Friedrich-Alexander-University Erlangen-Nürnberg, Hartmannstr. 14, 91052 Erlangen, Germany; Barbara.Ruder@uk-erlangen.de

**Keywords:** phagocytosis, macrophages, inflammation, intestine, IBD

## Abstract

Macrophages are part of the innate immunity and are key players for the maintenance of intestinal homeostasis. They belong to the group of mononuclear phagocytes, which exert bactericidal functions and help to clear apoptotic cells. Moreover, they play essential roles for the maintenance of epithelial integrity and tissue remodeling during wound healing processes and might be implicated in intestinal tumor development. Macrophages are antigen-presenting cells and secrete immune-modulatory factors, like chemokines and cytokines, which are necessary to activate other intestinal immune cells and therefore to shape immune responses in the gut. However, overwhelming activation or increased secretion of pro-inflammatory cytokines might also contribute to the pathogenesis of inflammatory bowel disease. Presently, intestinal macrophages are in the center of intense studies, which might help to develop new therapeutic strategies to counteract the development or treat already existing inflammatory diseases in the gut. In this review, we focus on the origin of intestinal macrophages and, based on current knowledge, discuss their role in the gut during homeostasis and inflammation, as well as during intestinal wound healing and tumor development.

## 1. Introduction

For a long time, the digestive tract was described to have an immense surface with 260–300 m^2^. More recently, the surface area was re-calculated to be “only” 32 m^2^ [1]. With this huge surface, the gut fulfills its necessary tasks, e.g., food digestion, as well as water and nutrient absorption. The intestine is outlined with a single-cell epithelial layer, which is organized in crypts and villi in the small intestine and long crypts in the large intestine to increase the absorptive surface [2]. Intestinal epithelial cells (IECs) are characterized by an enormous self-renewing capacity. They arise from stem cells at the crypt bottom and while differentiating into different types of epithelial cells (secretory or absorptive lineage) they migrate towards the villi tips where they are finally shed into the intestinal lumen after 4–5 days [2,3]. A strict balance between proliferation at the crypt bottom and cell removal via epithelial “shedding” is indispensable to maintain intestinal homeostasis. Increased proliferation might lead to the development of intestinal tumors and colorectal cancer, whereas increased cell death in the epithelium might induce inflammation and therefore might trigger the development of inflammatory bowel disease (IBD) [4]. The two main clinical forms of IBD are Crohn’s disease (CD) and ulcerative colitis (UC). They are chronically relapsing disorders, which cause several symptoms including, e.g., fatigue, diarrhea, weight loss, and pain. The exact underlying reasons for the development of IBD are not clear yet, however several factors, such as environmental and microbial factors, genetic susceptibility, and impaired immunity, are known to play a role for the pathogenesis of IBD [5,6,7].

Besides its function as a digestive organ, the gut can also be considered as the largest immune organ of the human body [8]. Beneath the intestinal epithelial cell layer which constitutes the primary barrier to stop invading pathogens, the gut harbors many different immune cells from both the adaptive and innate arm of the immune system [4,9]. These immune cells on the one hand fight against harmful invading bacteria to prevent inflammation, but on the other hand they also need to distinguish between beneficial and harmful factors to prevent excessive immune reactions. Moreover, they may not fight against commensal bacteria, which are needed for a proper function of the intestine [10]. Intestinal macrophages and other immune cells are supported by mucins and antimicrobial peptides which are secreted by goblet and Paneth cells in the intestinal epithelium to trap and oppose harmful bacteria [11]. Within the lamina propria, there are cells of the myeloid lineage which sense foreign antigens, either in lymphoid follicles beneath the follicle-associated epithelium or also along the normal villous epithelium [12]. They present foreign antigens to T- and B-lymphocytes, which subsequently induce a specific immune response to eliminate invading pathogens and to mediate antibody production [13,14,15]. A complex interplay between epithelial cells, innate and adaptive immune cells is therefore indispensable to shape the immune response in the gut and to prevent over-activation and auto reactivity [13].

The gastrointestinal mucosa harbors the largest number of macrophages in the body [16]. These intestinal macrophages play key roles for the maintenance of intestinal homeostasis, but also during inflammation, wound healing, infection, and tumorigenesis. Moreover, they are implicated in the development of IBD. Generally, macrophages have been classified into two subgroups. Classically activated M1-like macrophages, which are generated in response to tumor necrosis factor alpha (TNFα) or lipopolysaccharide (LPS) and interferon gamma (IFNγ), and alternatively activated M2-like macrophages, generated by stimulation with Interleukin (IL)-4 or IL-13 [17,18,19]. However, more recent studies suggest a more complex classification of macrophages based on their different activation stimuli [17,20]. Moreover, a new concept of macrophage classification based on their function was proposed by Mosser and colleagues in 2008. According to this model, macrophages can be divided into three different groups based on their homeostatic activities: host defense, wound healing, and immune regulation [21]. In this model, different cytokines produced by immune cells determine the developmental fate of these distinct macrophage populations. Classically activated macrophages play an important role for host defense reactions, are characterized by microbicidal activity, and arise by stimulation with IFNγ or TNFα, which can be produced by T cells, natural killer cells, or antigen-presenting cells. Wound healing macrophages, which are needed for tissue repair, develop in response to T cell or granulocyte produced IL-4 and regulatory macrophages, which exert anti-inflammatory activity, arise in response to several stimuli, e.g., IL-10, glucocorticoids, or apoptotic cells [21].

In this review, we focus on different populations of intestinal macrophages, their origin and development and their important roles in intestinal homeostasis and inflammation. Furthermore, we would like to discuss their influence on wound healing and intestinal tumor development. Since specific macrophage populations might contribute to the development of IBD, they potentially serve as a therapeutic target, which will also be addressed in this review.

## 2. Origin of Macrophages in the Gut

In the gut, macrophages are present directly beneath the intestinal epithelial cell layer in the lamina propria [22], but also in deeper layers of the gut, including the submucosa and the muscularis externa. Generally, in early studies, macrophages were reported to evolve from hematopoietic precursors in the bone marrow. From there, they are transported via the peripheral blood stream as monocytes to the target organ, where they finally differentiate into tissue macrophages (reviewed in [23,24]). The evolution of precursor cells in the bone marrow, their progression to monocytes in bone marrow and blood, as well as their subsequent migration into different tissues and differentiation into macrophages were altogether consolidated in a linear mononuclear phagocyte system [23]. However, more recent studies have challenged the concept of this mononuclear phagocyte system. Interestingly, not all tissue macrophages evolve in the bone marrow and finally migrate into different tissues. In contrast, several studies showed that tissue resident macrophages in embryos develop independently of hematopoietic stem cells and further persist in several adult tissues [25,26,27]. These findings were underlined, by showing that even after bone marrow transplantation, host macrophages could further expand, even if the donor macrophages were genetically compromised, suggesting that tissue-resident and circulating macrophages are part of different mononuclear phagocyte lineages, which expand independently from each other [28]. In 2012, Schulz and colleagues demonstrated that some macrophage populations develop in the embryonic Yolk-sac before hematopoietic stem cells (HSC) even exist. They further showed that expression of the transcription factor *Myb* is required for the development of HSC-derived macrophages, but is indispensable for Yolk-sac derived macrophage populations like liver Kupffer cells or epidermal Langerhans cells [26]. In 2015, Perdiguero and colleagues moreover showed that the majority of murine tissue macrophages in the liver, brain, epidermis, and lung develop from an erythro-myeloid progenitor, which gives rise to fetal macrophages early during embryonic development [25].

**Figure 1:**
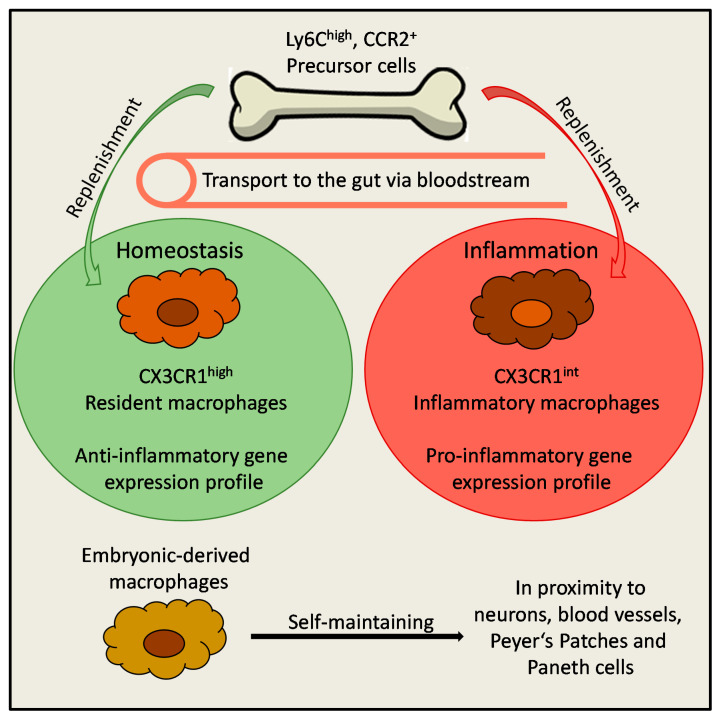
Development of macrophages in the gut. Precursor cells in the bone marrow express high levels of *Ly6C* and *Ccr2*, which are needed for their egress to the blood stream. Under homeostatic conditions, CX3CR1^high^ resident, anti-inflammatory macrophages develop from these precursor cells. However, under inflammatory conditions, this process is interrupted, leading to the accumulation of CX3CR1^int^ inflammatory macrophages. In addition to constantly replenished macrophages, also self-maintaining embryonic-derived macrophages are present in the gut. They are located in close proximity to neurons, blood vessels, Peyer’s patches and Paneth cells.

Then, how exactly is the situation in the gut, the biggest accumulation of macrophages in the body? In 2006, it was shown by Smythies et al. that circulating blood monocytes are attracted by the chemokines IL-8 and Transforming growth factor beta (TGFβ), which are constitutively produced by mucosal epithelial cells, and subsequently migrate to the lamina propria, underlining that blood monocytes constitute an important source of mucosal macrophages [29]. Subsequently, in 2012, the term “monocyte waterfall” was introduced [30]. In this study, Tamoutounour and colleagues demonstrated that gut-resident macrophages develop from extravasated Ly6C^high^ monocytes. These Ly6C^high^ monocytes express CCR2 (Chemokine Receptor 2), which is needed for their egression from the bone marrow to the blood [31] and undergo several intermediate developmental steps until they acquire typical features of intestinal macrophages [30] (Figure 1). Moreover, in 2014, Bain and colleagues demonstrated that during murine neonatal development, embryonic precursor cells settle in the intestinal mucosa where they extensively proliferate. Around weaning time of mice, these cells are replaced by a specific group of monocytes (Ly6C^high^) that derive from bone-marrow and blood and differentiate into mature macrophages with anti-inflammatory properties. This accumulation of colonic macrophages between the first two or three weeks after birth coincides with the acquisition of commensal microbiota. In line with these findings, it was shown, that in the colon, this process is mainly driven by the microbiota and needs to be continued throughout life to maintain the intestinal macrophage population [32]. Interestingly, in the small intestine, replenishment of macrophages from the blood is not altered in germ-free mice as compared to specific pathogen free (SPF) mice. In contrast to the colon, the process in the small intestine is rather dependent on dietary amino acids, suggesting a diet and not microbiota dependent regulation of small intestinal macrophages [33]. More recently, it was shown that beside this constantly replenished macrophage population, also other macrophage subsets exist in the gut, which differentially express the markers *Tim-4* and Cd4. In this study, TIM-4^+^ CD4^+^ macrophages were characterized by specific developmental dynamics and were found to be locally maintained in the gut, suggesting that not all macrophages in the adult intestine derive from constantly renewed precursor cells from the blood [34]. In line with these findings, in 2018, De Schepper and colleagues demonstrated that distinct groups of embryonic-derived macrophages remain in the gut even after weaning [35]. They are located in close proximity to enteric and myenteric neurons, blood vessels, Peyer’s patches, and Paneth cells. Interestingly, depletion of these self-maintaining embryonic-derived macrophages could be compensated by bone marrow- derived macrophages, which reconstituted the free niches in the adult intestine. Moreover, depletion of these embryonic-derived macrophages leads to increased neuronal apoptosis and altered morphology and abundance of blood vessels. These findings suggest that embryonic-derived macrophages influence enteric neurons and therefore regulate essential intestinal functions including secretion and motility. This study nicely demonstrated that embryonic-derived macrophages, together with constantly renewed blood-derived macrophages, play an important role for the maintenance of intestinal homeostasis [35]. Taken together, during the early stages of neonatal development, embryonic precursor cells settle in the gut and proliferate until the weaning of mice. Afterwards, these precursor cells are almost completely replaced by newly arriving precursors from the bone marrow, which subsequently develop into mature intestinal macrophages. This switch is driven by the microbiota in the colon or is diet-dependent in the small intestine and constitutes a special feature of the gut, which differs from other organs. In addition to this constant replenishment of resident intestinal macrophages, specific populations of embryonic-derived macrophages remain in the gut even after weaning, and these were shown to regulate enteric neuronal functions.

## 3. Function of Intestinal Macrophages and Maintenance of Tissue Homeostasis

Since the intestine harbors the greatest bacterial load in the human body [36], intestinal immune cells on the one hand are needed to fight against harmful invading pathogens and to prevent bacterial dysbiosis. On the other hand, they must tolerate beneficial probiotic bacteria to circumvent inadequate detrimental immune responses. Intestinal macrophages play an important role to maintain this balance. Interestingly, the intestinal microbiota shapes and regulates the pool of intestinal macrophages as demonstrated by the fact, that germ-free mice are characterized by reduced total numbers of intestinal macrophages and reduced numbers of specific colonic macrophage populations (CD11c^+^ CD206^int^ CD121b^+^ and CD11c^−^ CD206^hi^ CD121b^−^) [32,37], underlining the important cross-talk between the gut microbiota and macrophage function.

**Figure 2:**
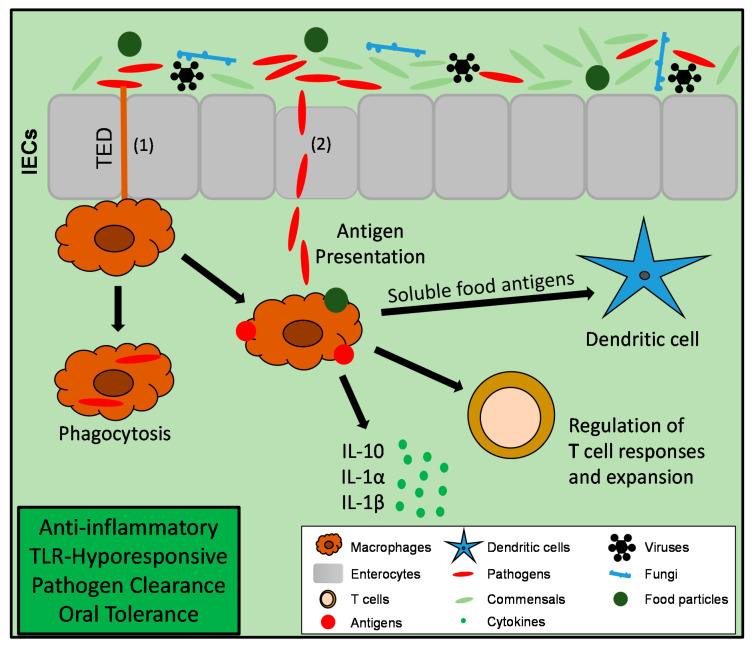
Macrophages in the intestine under homeostatic conditions. Macrophages take up bacterial antigens or food particles either with help of their transepithelial dendrites (TED, 1) or via the entry route through epithelial M-cells (2). Intestinal macrophages phagocytose and eliminate invading pathogens while at the same time preventing overwhelming immune responses due to their TLR-hyporensponsiveness. They present foreign antigens or food particles to dendritic cells and also T-cells, an important step to mediate T cell regulation and food tolerance. They are characterized by an anti-inflammatory gene expression profile with spontaneous expression of high amounts of *IL-10*, which plays a key role for the maintenance of intestinal homeostasis.

In general, macrophages are key elements of the intestinal immune system and are needed to maintain intestinal homeostasis. They belong to the group of mononuclear phagocytes and constitute an important innate defense line against invading pathogens. They eliminate invading microbes without triggering exaggerated immune reactions. For the recognition of harmful bacteria, they express several pattern recognition receptors, e.g., Toll-like receptors (TLRs) and NOD-like receptors (NLRs), which help them to distinguish between commensal and harmful bacteria [38,39,40]. Intestinal macrophages are highly phagocytic but do not upregulate pro-inflammatory cytokines after uptake of pathogens [41]. Characteristically, resident intestinal macrophages express only low levels of pro-inflammatory mediators, such as *IL-1a*, *IL-6*, *TNFA*, or inducible nitric oxide synthase (*iNOS*) [41,42,43,44]. In contrast, they spontaneously secrete high levels of IL-10, which was described to contribute to their hyporesponsiveness to TLR-stimulation [42,45,46,47,48,49,50]. Altogether, these characteristics indicate a rather anti-inflammatory profile of resident intestinal macrophages (Figure 2). Due to their hyporesponsiveness to TLR-stimulation and their secretion of anti-inflammatory cytokines, the onset of inflammation due to bacterial stimulation in the gut under steady state conditions is prevented [40,41].

In line with this view, under steady state conditions, mature colonic macrophages were shown to express genes that are involved in phagocytosis and uptake of apoptotic cells (*Mertk*, *Mrc1*, Cd36, *Gas6*, *Axl*, *Itgav*, *Itgb5*, Cd9, Cd81, *C1qa-c*) [51,52]. In a novel in vivo model of apoptosis induction in IECs under non-inflammatory conditions, two macrophage populations in the small intestinal lamina propria were shown to clear apoptotic IECs via phagocytosis. These two macrophage populations were identified based on their expression of specific surface markers (CD103^+^ CD11b^+^ and CD11b^+^). Upon sampling of apoptotic IECs, these macrophage populations significantly upregulated genes involved in the clearance of apoptotic cells, including *Gas6*, Cd163 and *Mertk*. Moreover, they expressed Pikfyve, a gene essential for phagosome maturation [53]. This study provided strong evidence that intestinal epithelial cells are not just shed from the villous tip to maintain constant epithelial cell numbers. In contrast, intestinal macrophages play an important role for the clearance of apoptotic cells and therefore influence the maintenance of epithelial barrier integrity under steady state conditions in the gut.

In 2001, it was described that pathogens not only penetrate the intestinal mucosa at M-cells, which are located in the follicle-associated epithelium of Peyer’s patches [54]. Instead, mononuclear phagocytes in the gut are able to sense and take up bacterial antigens in the intestinal lumen with the help of their transepithelial dendrites (TEDs) via opening of epithelial tight junctions [54,55]. These studies suggested multiple entry routes of bacterial pathogens in the gut. In 2005, these TED forming cells were identified as CX3CR1 (CX3C Chemokine Receptor 1)^high^ dendritic cells. After the discovery that CX3CR1 constitutes a specific marker on intestinal macrophages, it became clear that CX3CR1^high^ macrophages rather than dendritic cells take up bacterial antigens with the help of their TEDs [56,57,58,59] (Figure 2). Being in line with the fact that CX3CR1 is an important marker for intestinal macrophage homeostasis, *Cx3cr1^−/−^* mice were characterized by reduced numbers of lamina propria macrophages. Interestingly, in the same study, it was demonstrated that bacterial translocation was not driven by defective epithelial barrier function but rather by the lack of these lamina propria macrophages under steady state conditions [57]. Besides CX3CR1, it was also shown that its ligand CX3CL1 is necessary for TED formation of intestinal macrophages [58,60]. The question if lamina propria macrophages transport bacterial antigens to the mesenteric lymph nodes (MLNs) is still under debate. Schulz and colleagues demonstrated that CX3CR1^+^ cells do not migrate to MLNs under steady state and acute inflammatory conditions [61]. These findings were underlined by results from Diehl et al. showing that trafficking of luminal antigens under steady state conditions in the gut is indeed inhibited by the intestinal microbiota. Therefore, microbiota mediated signaling might limit immune priming against intestinal antigens to efficiently maintain tolerance towards commensal bacteria. However, under certain circumstances, e.g., in the absence of MYD88 or during antibiotics-mediated dysbiosis, CX3CR1^+^ mononuclear phagocytes do transport non-invasive bacteria to MLNs in a CCR7-dependent manner inducing T-cell responses and immunglobulin A (IgA) production [62]. These results were underlined by the study from Zigmond et al. showing that also under chronic inflammatory conditions CX3CR1^high^ macrophages gain migratory capacity and are present in lymph nodes [49]. These studies show that under steady state conditions macrophages do not migrate to MLNs. However, if the intestinal microbiota is altered or during chronic colitis, they are able to change their habits and invade MLNs. Besides their ability to migrate to MLNs under certain circumstances, it was shown that CX3CR1^high^ macrophages take up soluble food antigens and transfer them to CD103^+^ dendritic cells. This interaction was dependent on the gap junction molecule Connexin-43 and required membrane transfer, suggesting an important role for gap junctions for antigen presentation and induction of food tolerance [63,64].

**Figure 3:**
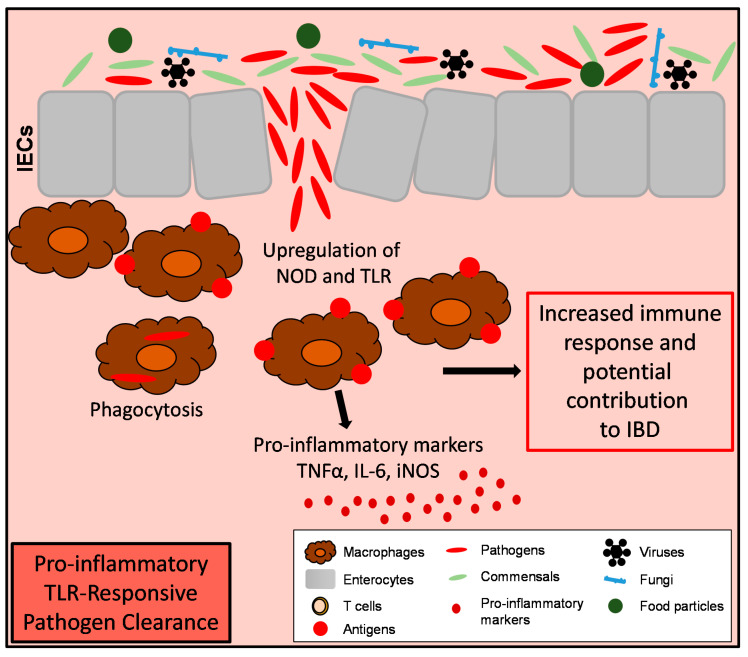
Macrophages in intestinal inflammation. Increased numbers of macrophages are present in the gut under inflammatory conditions. They respond to invading pathogens via expression of proinflammatory markers, like *TNFA*, *IL-6* and *iNOS*. These markers on the one hand regulate the immune response of other immune cells but might also perpetuate the development of inflammation in the gut. Therefore, inflammatory macrophages might display an important target for IBD therapy.

Beside their role in defense against invading pathogens via phagocytosis or uptake of bacterial antigens, intestinal macrophages also have the ability to cross-talk to other immune cells (Figure 2 and Figure 3). In addition to macrophages cross-talk with dendritic cells, as mentioned above [64], they are also described to play an important role for T-cell regulation in the gut under steady state conditions. Denning and colleagues showed that lamina propria macrophages induced IL-10 production of neighboring T-cells [45]. Moreover, IL-10 produced by lamina propria macrophages critically influences the regulation of CD4^+^ T-cell responses and Foxp3 (Forkhead Box P3)^+^ Treg (regulatory T) cell differentiation [45,65]. In accordance, IL-10 derived from CX3CR1^+^ lamina propria macrophages has been shown to be important for Foxp3 Treg cell expansion in the gut in a model of oral tolerance [46]. In 2012, it was furthermore described that IL-1β production by a subset of resident lamina propria macrophages is important for the development of T helper (Th) 17 cells in the gut under steady state conditions [66]. In 2014, Mortha and colleagues could show that intestinal macrophages are the highest producers of IL-1α and IL-1β in response to commensal microbiota, which is important to cross-talk to ILC3 (Group 3 innate lymphoid cells) and their Colony stimulating factor (CSF)-2 release, which in turn acted on macrophages and dendritic cells to maintain Treg homeostasis [67]. Moreover, in 2015 it was shown that CX3CR1^+^ mononuclear phagocytes prime T-cells and direct Th17 cell differentiation. In addition, CD64^+^ macrophages are essential to induce their response towards a specific microflora [68]. Collectively, these studies demonstrate an essential role of intestinal macrophages and their secreted cytokines for regulating T-cell responses in the gut.

Macrophage differentiation is mediated by CSF-1. This was demonstrated by *Csf-1* or *Csf-1* receptor (*Csfr*) knockout mice, which are mostly deficient for tissue macrophages [69,70,71,72]. Similarly, blocking CSF-1-signaling in adult mice by anti-CSFR antibody administration also almost completely depleted tissue macrophages, including intestinal macrophage populations [73]. Vice versa, administration of recombinant CSF-1 induced macrophage infiltration in tissues [74]. These studies already showed an important impact of CSF-1-signaling on macrophage homeostasis. Moreover, *Csf-1* expression of intestinal macrophages regulates the differentiation of intestinal epithelial cells. *Csf-1* expression in crypt-associated macrophages controls Paneth cell differentiation and indirectly disbalances the different stem cell populations in the crypt [75]. In addition, the blockade of CSF-1 signaling also impairs differentiation of other epithelial cell lineages, like goblet cells and M-cells [75], demonstrating an important role for macrophage signaling for the differentiation of epithelial cells and the maintenance of the intestinal homeostasis.

Besides their numerous important functions in the lamina propria, intestinal macrophages are also located distant from the intestinal lumen in deeper layers of the gut including the submucosa and the muscularis externa. These muscularis macrophages are characterized by a unique tissue specialization and reveal tissue-protective functions. Moreover, they express M2-associated genes like *Arg1*, *Chi3l3* and Cd163 [76]. They crosstalk to enteric neurons in a bidirectional manner. Enteric neurons communicate with this group of muscularis macrophages and constitutively produce CSF-1, a feature which is driven by microbial-derived signals and which is needed for maintenance and survival of these macrophages [77]. In addition, muscularis macrophages produce bone morphogenic protein (BMP)-2, which binds to its receptor on enteric neurons, contributing to correct neuronal function. This correlation was demonstrated by mice which were treated with a BMP-2-inhibitor resulting in altered intestinal contractility [77]. Beside these regulatory functions, under steady state conditions, muscularis externa macrophages also phagocytose dying neurons and neuronal debris in both small and large intestine [78]. In summary, these studies nicely show that this muscularis macrophage-neuron cross-talk regulates intestinal motility and neuronal function and that muscularis externa macrophages might actively regulate the enteric nervous system [76,77,79,80].

Anti-inflammatory, tissue resident macrophages are often classified as M2-like (alternatively activated) macrophages [81,82]. However, the current macrophage classification has been extended recently, suggesting a much higher number of different macrophage subgroups [83,84]. In a very recent study, single cell RNA-sequencing of mature colonic myeloid cells from SPF and germ-free (GF) mice was performed and identified seven different colon macrophage clusters, again challenging the existence of one classically defined macrophage population in the gut under steady state conditions [37].

## 4. Macrophages During Intestinal Inflammation

It became evident that mononuclear phagocytes and especially macrophages in the intestine can play important roles for the development of IBD [85,86]. In the mucosa of IBD patients, there is an accumulation of pro-inflammatory macrophages [87,88,89]. Moreover, also in pediatric IBD, increased numbers of activated mucosal macrophages were detected [90]. In general, these macrophages are characterized by increased expression of the pro-inflammatory markers *TNFa*, *IL-1b*, *IL-6*, and *iNOS* [88,91].

As mentioned above, under homeostatic conditions, resident macrophages in the murine colon (CX3CR1^high^) are highly phagocytic but resistant to TLR-stimulation and constitutively produce the anti-inflammatory cytokine IL-10 [41,47]. In addition to this group of macrophages, in the resting colon, there is also a smaller population of CX3CR1^int^ cells, which was shown to expand during experimental colitis in mice. Both groups (CX3CR1^high^ and CX3CR1^int^) derive from the same progenitor (Ly6C^high^ CCR2^+^ monocytes) (Figure 1). Once these CX3CR1^int^ macrophages arrive in the colon, they further develop into CX3CR1^high^ resident macrophages. In experimental colitis, this process is arrested, leading to an accumulation of inflammatory CX3CR1^int^ macrophages (Figure 1 and Figure 3). Analogous processes were also observed in human healthy mucosa and Crohn’s colitis [87]. In contrast to resident macrophages, these inflammatory CX3CR1^int^ macrophages are TLR-responsive [87] (Figure 3). Zigmond and colleagues showed that *Ccr2* expression is essential for the recruitment of Ly6C^high^ monocytes to the inflamed intestine and that these newly recruited monocytes upregulate TLR2 and NOD2, to increase responsiveness to bacterial factors to become pro-inflammatory effector cells. Depletion of these Ly6C^high^ pro-inflammatory cells ameliorated acute intestinal inflammation suggesting that these inflammatory macrophages might serve as potential targets for IBD-therapy [50]. These inflammatory macrophages are often classified as classically activated M1-like macrophages which are characterized by their pro-inflammatory gene expression profile (*TNFA*, *IL-6*, and *iNOS*) [21,82] (Figure 3). Of note, the resident CX3CR1^high^ macrophages keep their anti-inflammatory characteristics even if inflammatory macrophages are present in the gut at the same time [44,87].

Several factors have been described to play a role for the regulation of intestinal macrophages and their potential contribution to the development of IBD. In the following, we will further discuss the role of the CX3CR1/CX3CL (CX3C Motif Chemokine Ligand) 1 and IL-10/Signal Transducer And Activator Of Transcription (STAT) 3 signaling axis together with the proteins Gasdermin D and Triggering Receptor Expressed On Myeloid Cells (TREM) 1 for intestinal macrophage function and their influence on intestinal inflammation.

Since CX3CR1 is an important marker for intestinal macrophages, its impact on intestinal inflammation development has been analyzed, suggesting a possible role for CX3CR1/CX3CL1 for the pathogenesis of IBD. One study showed that loss of CX3CR1 or its ligand CX3CL1 rendered mice less susceptible to experimentally induced colitis [57]. In contrast, another more recent study showed that CX3CR1 and CX3CL1 were highly upregulated in the colon during experimentally induced colitis and that *Cx3cl1*-deficient mice and two different strains of *Cx3cr1*-deficient mice developed more severe colitis as compared to controls [92]. In accordance, polymorphisms of these genes in IBD patients were attributed to influence the clinical manifestations of IBD [93,94].

IL-10 and its receptor (IL-10R) were shown to play an important role for the maintenance of intestinal homeostasis as well as for the development of IBD [95,96,97,98,99,100,101,102]. Furthermore, it was shown, that IL-10/IL-10R-signaling is important for differentiation and function of intestinal macrophages in mice and IBD patients [103]. These findings were also underlined by the fact that the complete loss of IL-10 in a murine model mediated a shift of resident CX3CR1^high^ macrophages to pro-inflammatory macrophages in the colon [49]. Interestingly, *Il-10*-deficiency specifically in CX3CR1^high^ intestinal macrophages did not have any effect on gut homeostasis and regulation of regulatory T-cells [49]. However, loss of *Il-10r* expression in intestinal macrophages lead to a deregulation of mucosal immunity and drove the development of severe intestinal inflammation [49,104]. A very recent study further investigated the impact of IL-10R-signaling in macrophages on IL-23-signaling. Among others, IL-23 was characterized as an IBD susceptibility gene [105]. *Il-10ra*-deficient colonic macrophages showed a microbiota-dependent increased pro-inflammatory gene expression pattern, especially *Il-23* was upregulated. IL-23 in turn activated IL-22 production in both Th17 and ILC3 cells. Subsequently, IL-22 activated the antimicrobial peptide expression in IECs leading to induced neutrophil recruitment. Altogether, these events mediated the development of colitis induced by *Il-10ra*-deficient macrophages [104]. In summary, these studies all suggest a critical role for IL-10 and IL-10 receptor signaling for the development of intestinal inflammation. Among other cytokines, IL-10 can activate the downstream transcription factor STAT3. Interestingly, mice lacking STAT3 in myeloid cells (*LysMCre/Stat3fl/-*) were characterized by constitutive activation of macrophages in response to LPS, demonstrated by increased expression of *Tnfa* and *Il-6*. Moreover, *LysMCre/Stat3fl/-* mice showed impaired IL-10-mediated functions and enhanced Th1 activity, suggesting that STAT3 activation is indispensable for IL-10-mediated functions in myeloid cells. These findings were supported by the fact that these *LysMCre/Stat3fl/-* mice spontaneously develop enterocolitis, underlining the important role of the IL-10-STAT3 axis for maintaining immune tolerance and suppressing the development of intestinal inflammation [106].

Interestingly, a very recent study could show that Gasdermin D in macrophages is necessary to protect from exacerbated colitis in a murine model of experimentally induced colitis. Gasdermin D is an executioner of pyroptosis, which is a form of programmed cell death mediated by inflammasome activation. In macrophages, Gasdermin D was shown to inhibit the cGas (Cyclic GMP-AMP synthase)-STING (Stimulator of Interferon genes protein) pathway, which is described to trigger the activation of the transcription factors IRF3 (Interferon Regulatory Factor 3) and NFκB (Nuclear Factor kappa-light-chain-enhancer of activated B cells). These factors in turn activate the expression of pro-inflammatory cytokines, chemokines, and interferons, potentially driving inflammation. Altogether, this study suggests a protective function of the pyroptosis executioner Gasdermin D in macrophages during inflammation [107].

TREM1 is characterized as a strong enhancer of inflammatory responses in macrophages. It is not expressed on resident human lamina propria macrophages and also almost completely absent from intestinal mononuclear phagocytes from mice [44,108,109]. Downregulation of *Trem1* under steady state conditions might constitute another mechanism to suppress excessive overactivation of macrophages in response to the immense numbers of microorganisms present in the gut [108,109]. However, during experimentally induced colitis, the level of *Trem1* expression on inflammatory mononuclear phagocytes in mice is increased [44]. In line with these findings, the number of TREM1-expressing intestinal macrophages from the inflamed mucosa of IBD patients was also increased, mediating elevated expression of pro-inflammatory cytokines and chemokines. Interestingly, increased *TREM1*-expression also correlated with enhanced disease activity in these patients. Blocking of TREM1 in murine models of experimentally induced colitis ameliorated disease severity, suggesting TREM1 to be an attractive therapeutic target for IBD patients [109].

Interestingly, in 2010, it was shown that injection of M2-like macrophages ameliorated inflammation in the murine model of DNBS (dinitrobenzene sulfonic acid)-induced colitis, which was accompanied by increased IL-10 production. In contrast, classically activated M1-like macrophages did not protect from colitis in this model [85]. In line with these results, other studies further demonstrated a protection of mice from DSS-colitis by M2-like macrophages, implicating that M2-like macrophages exert anti-inflammatory functions in different mouse models of colitis [110,111,112]. Collectively, these studies led to the idea, that especially IL-4-conditioned M2-like macrophages from healthy patients can be used to treat IBD. Jayme et al. showed in a very recent study, that IL-4 treated human macrophages promoted epithelial wound healing in vitro and also reduced colitis in a mouse model in vivo. These results constitute a proof-of-concept study supporting the development of autologous IL-4-conditioned M2-like macrophages as a potential therapy for IBD patients [113]. Being in line with these studies, patients with active CD were characterized by reduced numbers of M2-like macrophages, whereas biopsies from patients with inactive CD revealed increased numbers [85].

Altogether, these studies again underline that intestinal macrophages and their polarization play an important role during inflammation both in murine models and also in IBD patients. Several factors implicated in macrophage function, like IL-10-signaling, TREM1 or Gasdermin D might display important target points for IBD therapy. Moreover, development of specific autologous macrophage populations might constitute an important tool for patient treatment. However, in recent years it has become crystal clear, that the classic discrimination between M1 and M2 macrophages is not fully recapitulated when isolating macrophages from tissue and macrophage polarization is far more complex in vivo than in vitro. Therefore, additional research is necessary to better understand the different polarization fates before autologous macrophages can be considered as a potential strategy for IBD therapy.

## 5. Intestinal Macrophages and Wound Healing

Beside their functions in intestinal homeostasis and inflammation, intestinal macrophages are also important for epithelial regeneration. These specific macrophages are either termed wound healing macrophages or are often classified as alternatively activated M2-like macrophages, more precisely M2a macrophages. These macrophages express factors that are important for epithelial regeneration and arise due to stimulation with IL-4 [21,114]. In 2005, Pull et al. could show that intestinal macrophages in the pericryptal stem cell niche transmit regenerative signals to neighboring colonic epithelial progenitors and promote epithelial healing after injury [115]. Especially TREM2-signaling plays an important role to drive epithelial wound healing via induction of cytokines that promote M2-like macrophage activation in the wound bed [116]. Moreover, in several studies it was shown that IL-10 produced by intestinal macrophages is an important mediator of epithelial wound healing [117,118]. Downstream of IL-10/IL-10R, CREB (cAMP response element-binding protein) is activated, which is subsequently followed by synthesis and secretion of WISP-1 (WNT1-inducible signaling protein 1). This CREB/WISP-1 axis is needed to activate epithelial proliferation and wound closure [118]. Of note, whereas, in the colon, epithelial regeneration is driven by the microbiota, which was shown by experiments using germ-free or MyD88-deficient mice, in the small intestine, microbiota depletion did not influence epithelial regeneration, suggesting different mechanisms of epithelial regeneration dependent on the intestinal segment [115,117]. In 2016, Cosín-Roger and colleagues demonstrated that the transcription factor STAT6 promotes M2-like polarization of macrophages, further driving the activation of WNT-signaling to promote mucosal repair in the murine model of TNBS (trinitrobenzene sulfonic acid)-induced colitis [119]. Another study could show that macrophages support epithelial regeneration by secretion of hepatic growth factor (HGF). Interestingly, macrophages derived from CD patients were characterized by reduced HGF secretion, potentially implicating epithelial regeneration in these patients [120]. In addition to these factors, the alarmin IL-33 was also recently reported to be co-localized with epithelial cells and F4/80^+^ cells in the lamina propria during oxazalone-induced colitis [121]. A subsequent study showed a direct effect of IL-33 on M2-like macrophage polarization and therefore positive effects on mucosal wound healing [122]. Interestingly, cobitolimod, a TLR9-agonist, reduced pro-inflammatory M1-like macrophages accompanied by reduced pro-inflammatory cytokine expression in vitro. At the same time, an increased number of M2-like wound healing macrophages and their *IL-10* expression was observed. Beside these in vitro observations, in the murine model of DSS-induced colitis, rectal cobitolimod treatment ameliorated intestinal inflammation in vivo, suggesting that TLR9 signaling influences macrophage polarization and mucosal wound healing [123]. Another recent study could show that impaired α4β7-dependent gut-homing of non-classical monocytes might mediate a reduction of wound healing macrophages, leading to impaired intestinal wound healing. Consequently, these results might initiate new studies to further investigate the role of Vedolizumab (anti α4β7-antibody) in IBD treatment and its effect on intestinal wound healing [124].

## 6. Macrophages and Intestinal Tumor Development

Tumor-associated macrophages (TAM) are described to affect several mechanisms involved in the formation of neoplastic tissue, e.g., tumor cell invasion, matrix remodeling, intravasation, seeding at distant sites and angiogenesis [125]. In their activated state, they are also described to elicit anti-tumor functions, therefore TAM can influence tumor growth in opposite directions (macrophage balance [126]). In general, whereas M1-like macrophages are suggested to act anti-tumorigenic during intestinal tumor development, M2-like macrophages are described to exert rather tumor-promoting functions. It was shown that the infiltration of M1-like macrophages in colorectal cancer (CRC) is followed by infiltration of M2-like macrophages, demonstrating the parallel appearance of both macrophage populations at the tumor front [127]. Moreover, the balance between the different macrophage populations might differ dependent on the disease stage and progression [128]. Cui and colleagues found that the M2/M1 ratio was increased in correlation to metastatic activity in the liver of CRC patients. More specifically, M1-like macrophages were negatively correlated with liver and lymph node metastasis and M2-like macrophages were positively correlated with the degree of tumor differentiation, as well as liver and lymph node metastatic ability [129]. These studies are supported by results from Nakanishi et al. who showed that in a murine model of sporadic tumor development (*ApcMin* mouse model) an elevated number of M2-like macrophages was observed in polyps as compared to surrounding tissue. Interestingly, the numbers of M2-like macrophages were even increasing with polyp size. Inhibition of cyclooxygenase 2 (COX-2) in this model reduced polyp size and mediated a change of macrophage polarization towards M1-like macrophages in an IFNγ-dependent manner, underlining the anti-tumor effects of M1-like macrophages and pro-tumorigenic effects of M2-like macrophages in intestinal tumorigenesis [130].

Beside these studies about the distribution of different macrophage populations in CRC, it was shown that in general, a high macrophage infiltration at the tumor front is accompanied with a positive prognosis of colon cancer [131,132,133] and inversely correlated to liver metastasis [133]. In contrast, Badawi and colleagues showed that macrophage infiltration was significantly higher in advanced tumor stages and correlated with lymph node metastasis [134]. In line with these results, another study showed that the pro-inflammatory markers produced by macrophages (TNFα, IL-1β, IL-6) induce proliferation and metastatic behavior in colon cancer cell lines [135]. Moreover, TAMs might induce elevated angiogenin expression in the tumor microenvironment, leading to increased angiogenesis, which constitutes a critical factor for tumor growth and metastasis [134,136]. Of note, other studies demonstrated that the number of infiltrating macrophages is decreasing in advanced tumor stages [137,138], suggesting that macrophages are less attracted to tumor cells in progressing CRC.

Altogether, these studies show diverse roles of TAMs in human CRC suggesting that the amount of infiltrating tumor macrophages and the exact function of the different macrophage populations is not fully elucidated yet. However clearly, TAMs are located in close proximity to tumor tissue, implicating an influence of these TAMs on intestinal tumorigenesis.

Interestingly, by using the murine *ApcMin* mouse model, Soncin and colleagues have recently shown that pro-tumorigenic macrophages can self-renew in the tumor tissue and maintain their numbers mostly independent from bone marrow derived invading progenitor cells. In this murine model, these intra-tumoral F4/80^+^ macrophages are CCR2-independent and promote tumor progression. These observations provide new insights into the characteristics of murine TAMs and might help to develop new therapeutic strategies [139].

Several molecules have attracted considerable attention regarding their impact on TAMs. In the following part, we would like to focus on the two proteins CSF-1 and GP96, which might constitute future target points for CRC therapy.

CSF-1 is described as a regulator of proliferation, differentiation and survival of macrophages and increased expression is found in several tumors (e.g., ovarian or endometrial adenocarcinomas) and correlates with high grade and poor prognosis [125,140,141]. In a co-culture experiment of colon cancer cells with macrophages, it was shown that a high amount of co-cultured macrophages induced higher levels of *CSF-1* expression and that high CSF-1 levels recruited higher numbers of macrophages vice versa [142]. In contrast to other organs, it was further shown that high CSF-1 levels and high macrophage infiltration were correlated with good prognosis and improved patient survival [142]. Additionally, this study showed that increased CSF-1 levels in colon cancer cells decreased the expression of pro-inflammatory markers in macrophages [142]. Reduced expression of these pro-inflammatory factors might further contribute to the suppression of colon tumor development [135,143]. However, data from the murine *ApcMin* tumor model suggest that CSF-1 supports maintenance and self-renewal of pro-tumorigenic macrophages in intestinal tumors, implicating rather tumor-promoting functions of CSF-1 in this mouse model [139].

The endoplasmatic reticulum chaperone GP96 was described to have important functions for macrophages in tumor development. Interestingly, in a murine model of experimentally induced colitis, *Gp96* knockout in gut-associated macrophages (*Gp96 x LysMCre*, *Mac96KO* mice) ameliorates inflammation. Moreover, in a murine model of colitis-associated colorectal cancer (AOM (Azoxymethane)/DSS)), these mice also developed fewer and less advanced tumors, potentially due to reduced activation of the canonical WNT-signaling pathway. These results were associated with reduced expression levels of specific pro-inflammatory markers (*Tnfa*, *Il-6*, *Ifng*, *Il-17*, and *Il-23*) in the colon tissue of *Gp96* knockout mice as compared to controls [144,145]. This study suggests that GP96 might display a potential target in intestinal macrophages to treat colitis-associated colorectal cancer.

## 7. Conclusions

In this review, we aimed to discuss the different roles of intestinal macrophages for the maintenance of intestinal homeostasis, but also for the development of intestinal inflammation, their influence on intestinal wound healing and their role during intestinal tumorigenesis. Most of the macrophages present in the adult gut are constantly replenished by Ly6C^high^ progenitor cells, which originally develop in the bone marrow. Under steady state conditions, resident intestinal macrophages (CX3CR1^high^) are highly phagocytic and are characterized by TLR-hyporesponsiveness to avoid overwhelming immune responses towards the plethora of microbes which are present in the gastrointestinal tract. These resident intestinal macrophages express only low levels of pro-inflammatory cytokines, but high levels of *IL-10*, which in turn influences other immune cells in the gut, e.g., expansion and regulation of T-cells. During inflammatory conditions, the propagation of CX3CR1^high^ cells is inhibited, resulting in CX3CR1^int^ cells that further differentiate into inflammatory macrophages. These macrophages are characterized by high expression of pro-inflammatory cytokines which further mediate inflammatory responses to fight against invading bacteria. However, these pro-inflammatory cytokines might also trigger exaggerated immune reactions, potentially driving inflammation. Therefore, intestinal macrophages might display attractive targets for the treatment of IBD patients. In addition to inflammation, intestinal macrophages are also described to play a role for intestinal wound healing and tumor development, generally underlining their important functions for health and disease in the gut. However, before intestinal macrophages can be considered as therapeutical targets for IBD or CRC patients, more detailed information about the different macrophage populations, which are present in the gut in different homeostatic and disease environments, as well as their diverse polarization statuses is absolutely indispensable. Therefore, additional studies are necessary until patients can fully benefit from macrophage targeting.
